# An Assessment of *GPX1* (rs1050450), *DIO2* (rs225014) and *SEPP1* (rs7579) Gene Polymorphisms in Women with Endometrial Cancer

**DOI:** 10.3390/genes13020188

**Published:** 2022-01-21

**Authors:** Magdalena Janowska, Natalia Potocka, Sylwia Paszek, Marzena Skrzypa, Kamila Żulewicz, Marta Kluz, Sławomir Januszek, Piotr Baszuk, Jacek Gronwald, Jan Lubiński, Izabela Zawlik, Tomasz Kluz

**Affiliations:** 1 Department of Gynecology and Obstetrics, Fryderyk Chopin University Hospital No. 1, 35-055 Rzeszow, Poland; gibala.magda@gmail.com (M.J.); sjanuszek4@gmail.com (S.J.); jtkluz@interia.pl (T.K.); 2 Laboratory of Molecular Biology, Centre for Innovative Research in Medical and Natural Sciences, College of Medical Sciences, University of Rzeszow, 35-959 Rzeszow, Poland; npotocka@ur.edu.pl (N.P.); sylwia.paszek@wp.pl (S.P.); mskrzypa@ur.edu.pl (M.S.); 20.kamilaa@gmail.com (K.Ż.); 3 Department of Pathology, Fryderyk Chopin University Hospital No. 1, 35-055 Rzeszow, Poland; marta.kluz@interia.pl; 4 Department of Genetics and Pathology, Pomeranian Medical University, 70-204 Szczecin, Poland; baszukpiotr@gmail.com (P.B.); jgron@pum.edu.pl (J.G.); lubinski@pum.edu.pl (J.L.); 5 Institute of Medical Sciences, College of Medical Sciences, University of Rzeszow, 35-959 Rzeszow, Poland

**Keywords:** selenoprotein, endometrial cancer, uterine cancer, gene polymorphism, glutathione peroxidase 1, *GPX1* rs1050450, Pro198Leu, type 2 deiodinase, *DIO2* rs225014, Thr92Ala, *SEPP1* rs7579

## Abstract

Background: Numerous studies indicate a relationship between the presence of *GPX1* (rs1050450), *DIO2* (rs225014) and *SEPP1* (rs7579) gene polymorphisms and the development of chronic or neoplastic diseases. However, there are no reports on the influence of these polymorphisms on the development of endometrial cancer. Methods: 543 women participated in the study. The study group consisted of 269 patients with diagnosed endometrial cancer. The control group consisted of 274 healthy women. Blood samples were drawn from all the participants. The PCR-RFLP method was used to determine polymorphisms in the *DIO2* (rs225014) and *GPX1* (rs1050450) genes. The analysis of polymorphisms in the *SEPP1* (rs7579) gene was performed by means of TaqMan probes. Results: There was a 1.99-fold higher risk of developing endometrial cancer in CC homozygotes, *DIO2* (rs225014) polymorphism (95% Cl 1.14–3.53, *p* = 0.017), compared to TT homozygotes. There was no correlation between the occurrence of *GPX1* (rs1050450) and *SEPP1* (rs7579) polymorphisms and endometrial cancer. Conclusion: Carriers of the *DIO2* (rs225014) polymorphism may be predisposed to the development of endometrial cancer. Further research confirming this relationship is recommended.

## 1. Introduction

Endometrial cancer is the most common cancer of the female reproductive system. In 2020, there were over 400,000 new cases of endometrial cancer worldwide, and an increase in the incidence compared to previous years was observed [1]. There are two types of endometrial cancer: I-estrogen-dependent and II-estrogen-independent. The main causes of endometrial cancer development include: obesity, hyperestrogenism, arterial hypertension, diabetes mellitus, inbreeding, early first menstruation, late menopause and anovulatory cycles [2].

Selenium is a trace element that is part of the selenoproteins. There are 25 selenoproteins in the human body. All of them contain selenium in their active center, which forms the amino acid selenocysteine (Sec) [3]. Scientific reports show the relationship between the level of selenium in the human body, the presence of appropriate polymorphisms in genes encoding selenoproteins and the risk of cancer development [4]. Selenoproteins have an antioxidant function. Due to the protection of cells against free radicals, they counteract carcinogenesis [5]. The polymorphisms selected in this study affect the activity of the encoded proteins and, consequently, numerous metabolic pathways in the human body.

The *GPX1* gene is located on the short arm of chromosome 3; it encodes the glutathione peroxidase 1 enzyme. This enzyme is mainly expressed in red blood cells, liver, lung and kidney tissues. It has a protective function against oxidative stress [6]. The most frequently described changes include the *GPX1* rs1050450 (Pro198Leu). As a result of this polymorphism, there is a change of cytosine (C) to thymine (T) in codon 198. This leads to the change of amino acids from proline (CCC) to leucine (CTC) [7].

The *DIO2* gene is located on the long arm of chromosome 14; it encodes the iodothyronine 2 deiodinase enzyme. This enzyme is found mainly in the thyroid gland, brain, pituitary gland, skeletal muscle, heart and skin [8]. The main function of DIO2 is the conversion of T4 to T3 thyroid hormones, and thus this enzyme is responsible for the correct amount and distribution of thyroid hormones [9]. The most frequently described polymorphisms in the iodothyronine 2 deiodinase gene include rs225014 (Thr92Ala). As a result of changing thymine (T) to cytosine (C) in codon 92, the amino acids are changed from threonine to alanine [10].

The *SEPP1* gene is located on chromosome 5 and encodes Selenoprotein P1 [11]. This protein is responsible for the transport of selenium in the body and has antioxidant functions [12]. It occurs mainly in the liver and plasma. One of the described *SEPP1* polymorphisms is rs7579, which changes guanine (G) to adenine (A) in the 3′ untranslated region (3′ UTR) of the mRNA [13].

It has recently been observed that some forms of selenoprotein genes are associated with the risk of several cancers. Such a relationship was observed between the rs1050450 polymorphism of the *GPX1* gene (glutathione peroxidase 1) and the following neoplasms: breast, prostate, bladder and lungs [14,15,16,17] and the rs7579 polymorphism of the *SEPP1* gene (selenoprotein P1) with colorectal and prostate cancer [18,19]. The rs225014 polymorphism of the *DIO2* gene (iodothyronine 2 deiodinase) was associated with the occurrence of diseases such as: type 2 diabetes, osteoporosis, Hashimoto’s disease, Graves’ disease, Alzheimer’s disease and autism [20,21,22,23]. Research on the carriage of these polymorphisms and the risk of developing endometrial cancer has not yet been conducted.

## 2. Materials and Methods

The study involved 269 patients diagnosed with endometrial cancer based on a previously performed histopathological examination. These patients reported to the Department of Gynecology and Clinical Obstetrics, Provincial Hospital No. 1, in Rzeszow in 2016–2020 in order to start oncological treatment. Before the planned surgery, blood samples were drawn from each patient. The blood was also collected from 274 healthy women who were included in the control group. All study participants agreed to have blood samples drawn for clinical trials. The research was approved by the Bioethics Committee (Resolution No. 90/B/2016 of the Bioethics Committee of the Regional Medical Chamber of 24 November 2016). All samples were stored at −80 °C until DNA isolation.

### 2.1. DNA Isolation

DNA from test and control groups was isolated from peripheral blood lymphocytes using the Quick Blood DNA Purification Kit (EURX, Gdańsk, Poland). The protocol attached by the manufacturer was followed. The quality and quantity of the isolated genomic DNA was determined using Nanodrop (Thermo Scientific, Waltham, MA, USA). All samples were standardized by dilution to a final concentration of 25 ng/μL. The samples were stored at −20 °C until further analysis.

### 2.2. Genotyping

The PCR-RFLP method was used to determine polymorphisms in the *DIO2* (rs225014) and *GPX1* (rs1050450) genes. The *DIO2* and *GPX1* polymorphisms were amplified using primers published by Xiong et al. The amplification was performed in the final concentration reaction mixture containing 1 × PCR buffer, 0.25 mM dNTP mixture, 1.5 mM MgCl2, Pc µM, each of the forward and reverse primers, 0.5 U Taq DNA polymerase (EURX, Gdańsk, Poland) and 50 ng of purified DNA. The final reaction volume was 20 µL. The PCR reaction conditions were as follows: initial denaturation at 95 °C for 10 min, extension of 35 cycles of denaturation at 95 °C for 30 s, hybridization at 58 °C for 45 s, extension at 72 °C for 45 s and final extension at 72 °C for 7 min. The reactions were performed in a T100™ thermocycler (Bio-Rad, Hercules, CA, USA). The PCR products were digested with the appropriate restriction enzymes and run on a 2% Midori Green Stain-stained agarose gel (Nippon Genetics, Düren, Germany).

The analysis of polymorphism in the *SEPP1* (rs7579) gene was performed by an allele discrimination test using the TaqMan genotyping test (G > A, test C___8806056_10—Thermo Fisher Scientific, Waltham, MA, USA). The real-time PCR reaction conditions were as follows: 95 °C for 10 min and 40 cycles, 95 °C for 15 sec and 60 °C for 1 min. The reaction was performed on a Roche Molecular Diagnostics Cobas z480 Analyzer in 96-well plates. The Endpoint Genotyping module (LightCycler 480 SW, version 1.5.1.62 SP2-UDF v.2.0.0, Roche, Pleasanton, CA, USA) was used for data analysis.

### 2.3. Statistics

In order to estimate the odds ratio (OR) of endometrial cancer depending on the genotype of the analyzed genes, one-way logistic regression models were used.

## 3. Results

The genotype distribution for all genes was at Hardy–Weinberg equilibrium. The allele frequencies for the *GPX1* gene were *p*(C) = 0.72 and *q*(T) = 0.28 in the study group and *p*(C) = 0.71 and *q*(T) = 0.29 in the control group, respectively. The allele frequency for the *DIO2* gene in the test group was *p*(T) = 0.61 and *q*(C) = 0.39, and in the control group, *p*(T) = 0.69 and *q*(C) = 0.31, whereas for the *SEPP1* gene in the test group, *p*(G) = 0.71 and *q*(A) = 0.29 and in the control group, *p*(G) = 0.72 and *q*(A) = 0.28; for all genes, *p* > 0.05.

Table 1 shows the frequency of genotypes of selected polymorphisms: *GPX1* (rs1050450), *DIO2* (rs225014) and *SEPP1* (rs7579) in the control and study groups.

In the case of the *GPX1* (rs1050450) polymorphism, the CC genotype was found in 139 patients from the control group and in 129 women from the study group. CT heterozygous was found in 108 women from the control group and 124 from the study group. A TT genotype was observed in 27 patients from the control group and 16 from the study group. The OR for the CT heterozygote was 1.24 (95% Cl 0.87–1.76), and for the TT homozygote was 0.64 (95% Cl 0.32–1.23). However, the differences observed were not statistically significant.

In the case of the *DIO2* (rs225014) polymorphism, 128 patients from the control group and 103 from the study group were TT homozygotes. TC heterozygotes were represented by 121 patients from the control group and 126 from the study group. CC homozygous was found in 25 patients from the control group and in 40 patients from the study group. A statistically significant, more frequent occurrence of the rs225014 polymorphism was found in patients with endometrial cancer compared to patients in the control group (*p*-value 0.017). The OR for the CC homozygote was 1.99 (95% Cl 1.14–3.53). The OR for the TC heterozygote was 1.29 (95% Cl 0.90–1.86), without, however, reaching statistical significance (*p*-value 0.2).

In the case of the *SEPP1* (rs7579) polymorphism, the distribution was as follows: 138 women from the study group and 145 from the control group had the GG genotype. A total of 107 women from the control group and 109 from the study group were heterozygous GA. An AA genotype was obtained in 22 patients from the test group and 22 from the control group. The odds ratio for the AA homozygote was 1.05 (95% Cl 0.55–1.99), and for the GA heterozygote was 1.07 (95% Cl 0.75–1.53). However, the results obtained are not statistically significant.

## 4. Discussion

Our study attempted to determine whether polymorphisms in selected genes important for selenium metabolism, *GPX1* (rs1050450), *DIO2* (rs225014) and *SEPP1* (rs7579), are associated with the risk of developing endometrial cancer. Significantly, one of the polymorphisms analyzed in the *DIO2* (rs225014) gene was more common in patients with endometrial cancer (*p*-value 0.017). The results of our study showed that carrying the CC genotype resulted in almost twice the risk of endometrial cancer (OR 1.99, 95% Cl 1.14–3.53). In the review of the literature, we did not find any papers with a similar research problem. The presence of TC polymorphism also indicates a trend that may be associated with an increased risk of endometrial cancer (OR 1.29), but these differences did not reach statistical significance (*p*-value 0.2) (Table 1). Our observations should be validated in a larger group of patients and in other populations in independent studies.

So far, increased activity of the *DIO2* gene has been observed in follicular, anaplastic and medullary thyroid cancer [24,25]. Similarly, increased expression of the *DIO2* gene was demonstrated in pituitary tumors and in brain gliomas [26,27]. Altered expression of the *DIO2* gene was also noted in skin cancer [28]. No correlation was observed between the occurrence of the *DIO2* (rs225014) polymorphism and neoplasms; however, numerous studies indicate a correlation between the *DIO2* (rs225014) polymorphism and chronic diseases. In 2010, it was shown that CC homozygote carriers had a higher risk of developing type II diabetes (OR 1.41, 95% Cl 1.03–1.94, *p*-value 0.03). Meta-analyzes confirmed this relationship—the total effect from four studies indicated an OR of 1.18 (95% Cl 1.03–1.36, *p*-value 0.02) [20]. It was also found that people with the CC genotype already diagnosed with type 2 diabetes had higher glycated hemoglobin (HbA1C) scores, which indicated poorer glycemic control in these patients [29]. In 2007, N. Grarup et al. discovered no effect of the *DIO2* (rs225014) polymorphism on the risk of developing type 2 diabetes, as well as on insulin resistance and obesity [30]. T. Ota et al. studied the influence of Thr92Ala polymorphism on obesity in children. An approximately 3.4 times higher risk of obesity was obtained in Ala/Ala homozygotes (95% Cl 1.498–7.687, *p*-value 0.003) compared to other genotypes [31]. The influence of the *DIO2* (rs225014) polymorphism on bone mineral density was also investigated. Research shows that people with Thr92Ala polymorphism have a higher risk of developing osteoporosis compared to people with the wild-type genotype [32,33]. In 2018, N. Inoue et al. analyzed the influence of the *DIO2* (rs225014) polymorphism on the occurrence of autoimmune thyroid diseases (Hashimoto’s disease and Graves’ disease). It was observed that patients with the TT and TC genotypes had an increased risk of developing autoimmune diseases of the thyroid gland, especially Hashimoto’s disease [21]. A. Guerra et al. found no effect of the Thr92Ala polymorphism on the occurrence of autoimmune diseases of the thyroid gland [34]. In 2018, the impact of the Thr92Ala polymorphism on the incidence of Alzheimer’s disease and dementia in the population of Americans of African and European origin was analyzed. It was observed that the *DIO2* (rs225014) polymorphism increased the risk of developing Alzheimer’s disease (AD)/dementia among African Americans (95% Cl 1.07–1.58, *p*-value 0.008) by 1.3 times. In the population of Americans of European descent, no influence of the *DIO2* gene polymorphism on the development of AD or dementia was reported [22]. In 2021, A.A. e Marcondes et al. studied the relationship between autism and the frequency of the Thr92Ala polymorphism. There was no correlation between the prevalence of a specific genotype and the prevalence of autism; however, it was noticed that people with a mutated genotype showed higher adaptive behavior compared to people with the wild-type genotype [23]. The observation of a potential relationship between the *DIO2* polymorphism (rs225014) and the incidence of endometrial cancer is the first consideration of this type.

The *GPX1* (rs1050450) polymorphism has been studied for the following cancers so far: breast, prostate, bladder, lung, head, neck and brain. In 2009, N.A. Ermolenkoa et al. observed that the presence of the T allele (rs1050450) in the *GPX1* gene reduces the risk of breast cancer (OR 0.74, 95% CI 0.58–0.94, *p*-value 0.012) [35]. In 2015, E. Jabłońska et al. conducted a study among Polish women diagnosed with breast cancer and showed that the presence of Pro/Leu and Leu/Leu reduced the risk of breast cancer (OR 0.61, 95% Cl 0.38–0.97, *p*-value 0.035) [36]. In 2010, J. Hu et al. performed a meta-analysis that showed that women of the African population with thymine instead of cytosine in codon 198 of the *GPX1* gene had an increased risk of developing breast cancer [37]. G. Ravn-Haren et al., in a case-control study of a Danish population of women, observed that patients with the CT or TT genotype of the *GPX1* (rs1050450) gene had a 1.43 times higher risk of breast cancer compared to patients with the CC genotype [14]. In 2004, D.G. Cox noted that the *GPX1* (rs1050450) polymorphism had no effect on the development of breast cancer [38].

In 2011, O. Erdem et al. reported no relationship between *GPX1* (rs1050450) polymorphisms and the risk of developing prostate cancer [39]. C. Kucukgergin et al. noticed that people with the Leu/Leu or Pro/Leu genotype had a higher risk of prostate cancer compared to men with the Pro/Pro genotype [15]. In 2013, T. Men et al. performed a meta-analysis of studies on the impact of the rs1050450 C > T polymorphism on the incidence of prostate cancer and observed no relationship between the polymorphism mentioned above and the development of prostate cancer [40].

In 2016, K. Hadami et al. showed no correlation between the *GPX1* (rs1050450) polymorphism and the incidence of bladder cancer [41]. Ch. Wang et al. performed a meta-analysis that showed that the rs1050450 C > T polymorphism was associated with the development of bladder cancer (nonCC versus CC: OR 1.72, 95%CI 1.09–2.70, *p*-value 0.019) [16]. In 2013, M. Cao et al. performed a meta-analysis, which also showed that the *GPX1* (rs1050450) polymorphism was associated with the occurrence of bladder neoplasms (nonCC versus CC: OR 1.876, 95% Cl 1.011–3.480, *p*-value <0.001) [42].

The impact of the Pro198Leu polymorphism was also studied to assess the risk of developing colorectal cancer. No such dependency was found. The only thing that was observed was that people with the TT genotype (rs1050450) who smoked cigarettes or drank alcohol had a higher risk of developing colorectal cancer (OR 2.56, 95% Cl 0.99–6.61, *p*-value 0.02 and OR 1.45, 95% Cl 1.17–1.81, *p*-value 0.02) compared to people without these addictions [43,44].

In 2008, A. Rosenberger et al. conducted an analysis that showed that the carriers of the Leu *GPX1* allele (Pro198Leu) had a reduced risk of developing lung cancer (OR 0.6, 95% Cl 0.4–0.8, *p*-value 0.002 and OR 0.3, 95% Cl 0.1–0.8, *p*-value 0.012 for heavy smokers) [45]. Studies from 2007 also showed that people with the TC or TT genotype were less likely to develop lung cancer compared to patients with the CC genotype [46]. The opposite conclusions were reached by D. Ratnasinghe et al. Their research showed that people with the TC and TT genotypes had a higher risk of developing lung cancer compared to those with the wild-type genotype (OR 1.8 and OR 2.3, respectively) [17].

A meta-analysis from 2017 showed a connection between the rs1050450 C > T polymorphism and the development of head, neck and brain cancer [16].

The *SEPP1* (rs7579) polymorphism was analyzed for the colon and prostate. In 2010, C. Méplan et al. reported that people with the nonGG genotype of the *SEPP1* (rs7579) polymorphism had a 1.43 times higher risk of developing colorectal cancer compared to patients with the GG genotype (95% Cl 1.00–2.05, *p*-value 0.048). The OR for AA homozygotes was 2.47 (95% Cl 1.16–5.23, *p*-value 0.019) [18]. In 2013, an analysis of European case-control studies was performed, and it showed that AA homozygotes of the *SEPP1* (rs7579) polymorphism had a 1.67 times higher risk of developing colorectal cancer [19]. In 2019, G. Amini et al. reported an OR 1.63 (95% Cl 0.99–2.07, *p*-value 0.05) for the presence of the A allele in the rs7579 *SEPP1* polymorphism [47]. In 2010, A. Sutherland et al. found no correlation between the *SEPP1* (rs7579) polymorphism and the development of colorectal cancer [48].

An increased risk of prostate cancer development was observed in patients with the AA genotype (OR 1.72, 95% Cl 0.99–2.98) [19]. In 2010, in a cohort study of 248 patients diagnosed with prostate cancer and 492 patients in the control group, the risk of developing prostate cancer was 1.72 times higher in patients with known *SEPP1* (rs7579) polymorphism, but without statistical significance (*p*-value 0.22) [49].

However, our analysis did not show a relationship between *GPX1* (rs1050450) and *SEPP1* (rs7579) and the occurrence of endometrial cancer.

In addition, in our research, apart from selected polymorphisms, we analyzed the most common risk factors for endometrial cancer: obesity, diabetes and arterial hypertension. We subjected the results to univariate and multivariate analysis.

In 2017, D. Aune et al. conducted a literature review and meta-analysis of the research. From 19 case-control studies and 6 cohort studies, the total relative risk (RR) of developing endometrial cancer in patients with arterial hypertension was 1.61 (95% CI 1.41–1.85) [50]. O. Raglan et al. reported a relationship between BMI and the development of endometrial cancer where the OR for BMI was 1.21 (95% CI 1.13–1.29) [51]. In 2014, C. Liao et al. performed a meta-analysis of patients with diagnosed diabetes. From the 29 studies, the cumulative relative risk of developing endometrial cancer in patients diagnosed with diabetes was 1.89 (95% CI 1.51–1.71, *p*-value < 0.001) [52].

Hypertension and obesity in both univariate and multivariate analyses increase the risk of endometrial cancer (*p*-value < 0.001). Diabetes mellitus in univariate analyses significantly influences the development of endometrial cancer (OR 2.86). However, there is no statistical relevance in multivariate analyses.

In the case of the *DIO2* polymorphism (rs225014) for the CC genotype, we obtained an OR of 1.99 in univariate analyses (95% Cl 1.14–3.53, *p*-value 0.017). In multivariate analyses for the same genotype, the OR was 2.06 (95% Cl 1.13–3.80, *p*-value 0.019). For the remaining TT and TC genotypes, statistical significance was not achieved in either univariate or multivariate analyses.

For the remaining analyzed polymorphisms, *GPX1* (rs1050450) and *SEPP1* (rs7579), statistical significance was not achieved in either univariate or multivariate analyses (Table 2).

## 5. Conclusions

In the literature review, no analyses were found regarding the relationship between the polymorphisms *GPX1* (rs1050450), *DIO2* (rs225014) and *SEPP1* (rs7579) and the possibility of developing endometrial cancer. Our analyses show that the *DIO2* (rs225014) polymorphism may predispose to the development of endometrial cancer. Due to the fact that no similar studies were found during the literature review, and our studies were conducted on a limited number of patients, further analyses are recommended to confirm the relationship between the *DIO2* (rs225014) polymorphism and the development of endometrial cancer.

## Figures and Tables

**Table 1:** The frequency of genotypes, the odds ratio and 95% confidence thresholds for selected gene polymorphisms: *GPX1*, *DIO2*, *SEPP1*.

Genotypes	Controls = 274 ^1^	Cases = 269 ^1^	OR ^2^	95% CI ^2^	*p*-Value
*GPX1* (rs1050450)					
CC	139 (51%)	129 (48%)	—	—	
CT	108 (39%)	124 (46%)	1.24	0.87–1.76	0.2
TT	27 (9.9%)	16 (5.9%)	0.64	0.32–1.23	0.2
*DIO2* (rs225014)					
TT	128 (47%)	103 (38%)	—	—	
TC	121 (44%)	126 (47%)	1.29	0.90–1.86	0.2
CC	25 (9.1%)	40 (15%)	1.99	1.14–3.53	0.017
*SEPP1* (rs7579)					
GG	145 (53%)	138 (51%)	—	—	
GA	107 (39%)	109 (41%)	1.07	0.75–1.53	0.7
AA	22 (8%)	22 (8%)	1.05	0.55–1.99	0.9

^1^ n (%); ^2^ OR = odds ratio, CI = confidence interval.

**Table 2:** Univariate and multivariate analysis of selected polymorphisms and endometrial cancer risk factors.

	Univariable Logistic Regression			Multivariable Logistic Regression		
Characteristic	OR ^1^	95% CI ^1^	*p*-Value	OR ^1^	95% CI ^1^	*p*-Value
*GPX1* (rs1050450)						
CC	—	—		—	—	
CT	1.24	0.87–1.76	0.2	1.18	0.80–1.72	0.4
TT	0.64	0.32–1.23	0.2	0.68	0.33–1.36	0.3
*DIO2* (rs225014)						
TT	—	—		—	—	
CC	1.99	1.14–3.53	0.017	2.06	1.13–3.80	0.019
TC	1.29	0.90–1.86	0.2	1.32	0.90–1.95	0.2
*SEPP1* (rs7579)						
GG	—	—		—	—	
AA	1.05	0.55–1.99	0.9	1.17	0.58–2.34	0.7
GA	1.07	0.75–1.53	0.7	1.23	0.84–1.81	0.3
Hypertension						
No	—	—		—	—	
Yes	3.18	2.25–4.54	<0.001	2.33	1.59–3.42	<0.001
Diabetes						
No	—	—		—	—	
Yes	2.86	1.76–4.75	<0.001	1.85	1.08–3.21	0.027
Obesity						
No (<30 BMI)	—	—		—	—	
Yes (≥30 BMI)	3.08	2.15–4.45	<0.001	2.36	1.61–3.49	<0.001

^1^ OR = odds ratio; CI = confidence interval.

## Data Availability

The data presented in this study are available on request from the corresponding authors.
